# Supplementary material for: FpnA, the Aspergillus fumigatus homolog of human ferroportin, mediates resistance to nickel, cobalt and gallium but does not function in iron homeostasis
Source: Commun Biol. 2025 Mar 8;8:399. doi: 10.1038/s42003-025-07799-1 (PMC11890741; doi:10.1038/s42003-025-07799-1)
Supplement: Supplementary file 1 — Supplementary Information [file 42003_2025_7799_MOESM1_ESM.pdf]

Supplementary Information for

FpnA, the *Aspergillus fumigatus* homolog of human ferroportin, mediates resistance to nickel, cobalt and gallium but does not function in iron homeostasis

Isidor Happacher[1§], Simon Oberegger[1§], Beate Abt[1], Annie Yap[1], Patricia Caballero[1], Mario Aguiar[1], Javeria Pervaiz[1], Giacomo Gariglio[2], Matthias Misslinger[1], Clemens Decristoforo[2], Hubertus Haas[1*]

This includes:

Supplementary Fig. S1 | Annotated (Afu5g12920) and corrected (Afu5g12920-T) *fpnA* gene model in FungiDB JBrowse genome browser. (A) According to transcriptome data, the corrected *fpn1* gene model contains 5 typically small introns instead of the bioinformatically predicted two introns of which the second is unusually long. The screenshot of FungiDB JBrowse genome browser was taken on 25.05.2024. (B), (C) and (D) show the corrected genomic *fpnA* region (intron sequences in red), the *fpnA* coding region and the FpnA protein sequence, respectively.

Supplementary Fig. S2 | Nickel supplementation and *fpnA* deletion, but not *fpnA* overexpression, decreases expression of urease encoding *ureB*. For Northern analysis, fungal strains were grown for 18 h at 37 °C in liquid minimal medium cultures using 20 mM urea as a sole nitrogen source, 1% xylose for induction of the P*xylP* promoter without (-) and with (+) nickel supplementation. The *gpdA* transcript levels and ethidium bromide-stained ribosomal RNA (rRNA) are shown as controls for loading and quality of RNA.

Supplementary Fig. S3 | Nickel supplementation enhances reddish coloration of mycelia (bottom view), which is indicative for increased urease activity and *fpnA* overexpression decreases reddish coloration of mycelia without (-) but not with (+) nickel supplementation. Fungal strains were spot-inoculated on solid minimal medium containing 20 mM urea and 5 mM nitrate as nitrogen sources, 1% xylose for induction of the P*xylP* promoter, 0.012 g/L phenol red as a pH indicator without (-) and with (+) nickel supplementation. Plates were incubated at 37 °C for 72 h. The top view is shown only as a control.

Supplementary Fig. S4 | Structural alignments of Fpn1 and FpnA show high similarities and highlight the protein differences found in two TM loops. (A) Alignment of the human Fpn1 structures from electron microscopy (EM) in gray with the hepcidin ligand (magenta) without assisting Fab45D8 (PDB DOI: 6WBV; https://doi.org/10.2210/pdb6wbv/pdb)[1] and the AlphaFold[2] prediction AF-Q9NP59-F1 in blue: DALI Z-score of 51.3[3]. (B) Structural alignment of the human Fpn1 AlphaFold prediction AF-Q9NP59-F1 (blue) and *A. fumigatus* FpnA calculated with ColabFold[4] (forest-green): DALI Z-score of 28.4; the human Fpn1 EM structure and the *A. fumigatus* FpnA ColabFold prediction show a DALI Z-score of 26.3 (structural alignment not shown). (A and B) The two loops in human Fpn1 that differ significantly compared to the *A. fumigatus* FpnA (also see Fig. 6) are not modelled in the EM structure (6WBV) but in the AlphaFold prediction; the loops are indicated in yellow (between TM6 and TM7: ^{267}GVKDSNIHELEHEQEP^{282}) and in firebrick red (between TM9 and TM10: ^{397}PLDLSVSPFEDIRSRFIQGESITPTKIPEITTEIYMSNGSNSANIVPETSP^{447}).

Supplementary Fig. S5 | Schematical illustration of genetic manipulations at the *fpnA* locus in the generated *fpnA* strains.

Supplementary Table S1 | FpnA homologs from selected species included in the phylogenetic analysis (Fig. 5). Given are the corresponding accession numbers from NCBI[5], E-values, query coverage, Bit-scores and taxonomy. The accession number of the query sequence of *Aspergillus fumigatus* FpnA from FungiDB that was used for blastp is highlighted in bold.

Supplementary Table S2 | Primers used in this study. Small letters represent primer overhangs designed for the construction of the plasmids. PC, plasmid construction; TCA, transformation cassette amplification; NB, Northern blot probe.

Supplementary Table S3 | Strains used in this study.

Supplementary Fig. S6 | Uncropped blot images from Fig. 2 and Supplementary Fig. S2.

Supplementary References

A

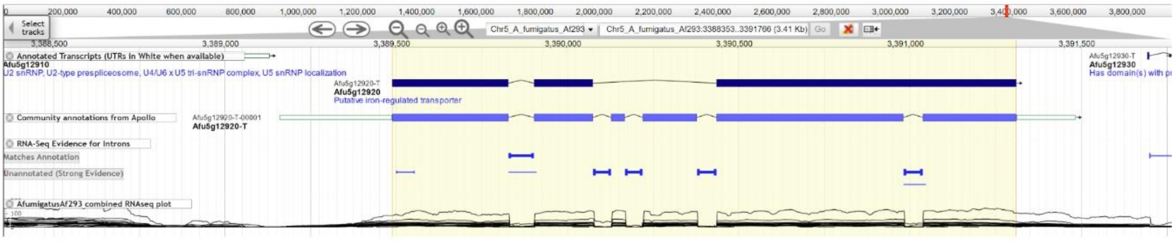

B **Genomic *fpnA* region**

ATGGCATCGCAGGTAGATATGAATAGTCACCAGGAAGACCTCCTGGAACCAACTGAAGCTCTTCTAGAAAGAGAAGCTCCCCCTGACGTACGGACAA
CTCGGACGGCAAGCTCTCGAAGTGTGTTGATAAGAATTTATGTCTCTCATTTCTTGTCGACATGGAACTCACGCATGTTTGAATTCGGCGCTGTCTTGT
TCCTTGCTTCTATCTTCCAGGGGACTCTGTTGTACGCCTCGATCTACGCGTTGGTACGCTCTGCGTCGGCGGTGGTTCTATCTTCCTGGTTAGGATCC
AAGATGGATCGGTCGAACCGTCTGGTAGCCATCCGTCACTCTATCGgtaggtcgcttctcctgcgacatactcccgctaaattaataatggatggctacatgacgg**cttac**acttcttagTC
TGGCAGAGAGTACCTGTTGCGGTGTCCTGCGCATGCTTTGTGGCTCTTCTGATGCCTTCATTCCGAGAATCTGAGCCTCTTGCCTCGGGCTTATTTCT
TGCGGTTGTCCTGCTCGCTTGCATGGAGAAACTGGCAGCAACAGCAAATACTGTGGCTGTCGAAAGAGACTGGgtaattcgtaacagtgaatacttctgggcaaaaa
aatt**ctaac**gccgggttagGTCATCATTGTTGCAGACACTTTAGCAATCGAGAGACAAGgtaggcaacctaacatactgatgttattcacgttagattgacgctgtaatagATTTGAATGC
ATCAATGAGACGGATAGATCTATTCTGCAAGCTTGTGGCCCCTGTGGTCATATCGCTCGTCGATGGCTTGTCTACAAAGGTCGCGGTCTGGACGGTCT
TGGGAGTGAATGCTCTTTCTGTCTTTATTGAATATATTGCCATTGCTCAGgtgagtttatccttttcgccctttacatcagtctgat**ctgac**cgggtattatgttagGTGTACAACGCGA
TACCAGAACTTGTGCGGACAGCTTCAGTCCCGACAGATGATGGATTCAGAGCTGTCGAGGATGCGCGCGAACATCCCACGGCGAACCAAAAGGGTA
TCATTAAAAGTGCAATTCACCTTGTAAAGCGGGCTGCTTCTCCCTGGCGACAGTATGTGGCTAGTCCAGTCTTCCTGGCGTCCTTCGCCTTGAGCCT
GCTCTATCTTACTGTTCTCTCCTTTGGCACCACCATGGTCACATATCTCTTGCATATGGGTTTTGATCCTCTGCAGGTCAGCTGTATGAGAATCGGTGC
CGTGCTCGCAGAATTGTCTGGCACATGGGCAGCACCGTTCATCATGGGCAGGATCGGACCGATTAGGTCAGGGCTGTGGTTCCTCAACTGGCAGCT
TGGCTGTTTGGCCACTGCAGCAGTCGCTTTTGCCTTGTACGACTCGAACTCTCGGTTGGTAGCAGTGAGTCTCATCCTGGGAGTTGCTTTGAGTCGG
ATTGGCCTTTGGGGTTTTGATTTGTCGGTTCAATTTCTCGTCCAAGAGgtaatcactttctcctctgagggctgctaccagaggttgctaatgtctatcaataagGGCGTTGAAGAA
GATACCCGGGGGCGATTCTCCTCAACCGAAATGGGCGTACAGAACGTCTTTGAGATGCTGTCGTTTGCAACCACAGTAGTCTTTCCTCTCCCTGAGC
AATTCAAGTATCCCGTTTTTATCAGTTACGGGGCGATTGCACTGGCAGCCATATGCTTTGCGGCATACGTGAGAAAAGAGCGGGGACATTTGCTGCAC
ATATCCAGGTGCTGGGGTGGTGATAAAATGCGAAGGTCGTATCAGGTACTTCCTGGAGGGTTATGA

C **Coding *fpnA* sequence**

ATGGCATCGCAGGTAGATATGAATAGTCACCAGGAAGACCTCCTGGAACCAACTGAAGCTCTTCTAGAAAGAGAAGCTCCCCCTGACGTACGGACAA
CTCGGACGGCAAGCTCTCGAAGTGTGTTGATAAGAATTTATGTCTCTCATTTCTTGTCGACATGGAACTCACGCATGTTTGAATTCGGCGCTGTCTTGT
TCCTTGCTTCTATCTTCCAGGGGACTCTGTTGTACGCCTCGATCTACGCGTTGGTACGCTCTGCGTCGGCGGTGGTTCTATCTTCCTGGTTAGGATCC
AAGATGGATCGGTCGAACCGTCTGGTAGCCATCCGTCACTCTATCGTCTGGCAGAGAGTACCTGTTGCGGTGTCCTGCGCATGCTTTGTGGCTCTTC
TGATGCCTTCATTCCGAGAATCTGAGCCTCTTGCCTCGGGCTTATTTCTTGCGGTTGTCCTGCTCGCTTGCATGGAGAAACTGGCAGCAACAGCAAAT
ACTGTGGCTGTCGAAAGAGACTGGGTCATCATTGTTGCAGACACTTTAGCAATCGAGAGACAAGATTTGAATGCATCAATGAGACGGATAGATCTATTC
TGCAAGCTTGTGGCCCCTGTGGTCATATCGCTCGTCGATGGCTTGTCTACAAAGGTCGCGGTCTGGACGGTCTTGGGAGTGAATGCTCTTTCTGTCT
TTATTGAATATATTGCCATTGCTCAGGTGTACAACGCGATACCAGAACTTGTGCGGACAGCTTCAGTCCCGACAGATGATGGATTCAGAGCTGTCGAG
GATGCGCGCGAACATCCCACGGCGAACCAAAAGGGTATCATTAAAAGTGCAATTCACCTTGTAAAGCGGGCTGCTTCTCCCTGGCGACAGTATGTGG
CTAGTCCAGTCTTCCTGGCGTCCTTCGCCTTGAGCCTGCTCTATCTTACTGTTCTCTCCTTTGGCACCACCATGGTCACATATCTCTTGCATATGGGTT
TTGATCCTCTGCAGGTCAGCTGTATGAGAATCGGTGCCGTGCTCGCAGAATTGTCTGGCACATGGGCAGCACCGTTCATCATGGGCAGGATCGGAC
CGATTAGGTCAGGGCTGTGGTTCCTCAACTGGCAGCTTGGCTGTTTGGCCACTGCAGCAGTCGCTTTTGCCTTGTACGACTCGAACTCTCGGTTGGT
AGCAGTGAGTCTCATCCTGGGAGTTGCTTTGAGTCGGATTGGCCTTTGGGGTTTTGATTTGTCGGTTCAATTTCTCGTCCAAGAGGGCGTTGAAGAA
GATACCCGGGGGCGATTCTCCTCAACCGAAATGGGCGTACAGAACGTCTTTGAGATGCTGTCGTTTGCAACCACAGTAGTCTTTCCTCTCCCTGAGC
AATTCAAGTATCCCGTTTTTATCAGTTACGGGGCGATTGCACTGGCAGCCATATGCTTTGCGGCATACGTGAGAAAAGAGCGGGGACATTTGCTGCAC
ATATCCAGGTGCTGGGGTGGTGATAAAATGCGAAGGTCGTATCAGGTACTTCCTGGAGGGTTATGA

D **FpnA Protein (509 aa)**

MASQVDMNSHQEDLLEPTEALLEREAPPDVRTTRTASSRSVLIRIYVSHFLSTWNSRMFEFGAVLFLASIFQGTLLYASIYALVRSASAVVLSSWLGSKMDRS
NRLVAIRHSIVWQRVPVAVSCACFVALLMPSFRESEPLASGLFLAVVLLACMEKLAATANTVAVERDWVIIVADTLAIERQDLNASMRRIDLFCKLVAPVVISLV
DGLSTKVAVWTVLGVNALSVFIEYIAIAQVYNAIPELVRTASVPTDDGFRAVEDAREHPTANQKGIIKSAIHLVKRAASPWRQYVASPVFLASFALSLLYLTVLS
FGTTMVTYLLHMGFDPLQVSCMRIGAVLAELSGTWAAPFIMGRIGPIRSGLWFLNWQLGCLATAAVAFALYDSNSRLVAVSLILGVALSRIGLWGFDLSVQFL
VQEGVEEDTRGRFSSTEMGVQNVFEMLSFATTVVFPLPEQFKYPVFISYGAIALAAICFAAYVRKERGHLLHISRCWGGDKMRRSYQVLPGGL

Supplementary Fig. S1 | **Annotated (Afu5g12920) and corrected (Afu5g12920-T) *fpnA* gene model in FungiDB JBrowse genome browser.** (A) According to transcriptome data, the corrected *fpn1* gene model contains 5 typically small introns instead of the bioinformatically predicted two introns of which the second is unusually long. The screenshot of FungiDB JBrowse genome browser was taken on 25.05.2024. (B), (C) and (D) show the corrected genomic *fpnA* region (intron sequences in red), the *fpnA* coding region and the FpnA protein sequence, respectively.

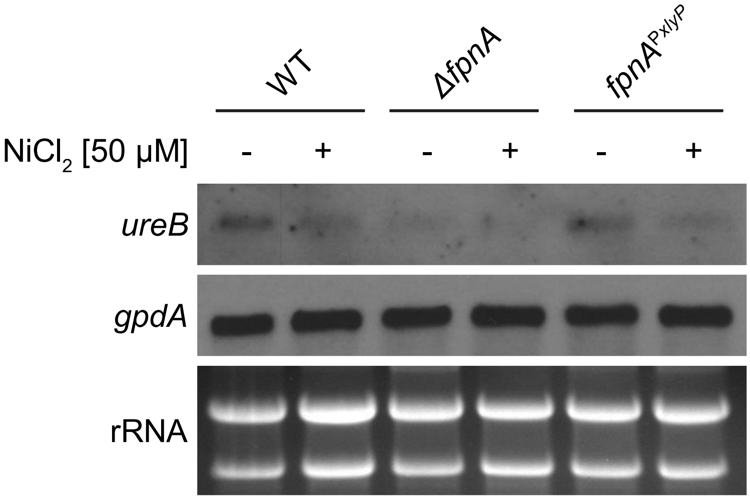

Supplementary Fig. S2 | Nickel supplementation and *fpnA* deletion, but not *fpnA* overexpression, decreases expression of urease encoding *ureB*. For Northern analysis, fungal strains were grown for 18 h at 37 °C in liquid minimal medium cultures using 20 mM urea as a sole nitrogen source, 1% xylose for induction of the P*xylP* promoter without (-) and with (+) nickel supplementation. The *gpdA* transcript levels and ethidium bromide-stained ribosomal RNA (rRNA) are shown as controls for loading and quality of RNA.

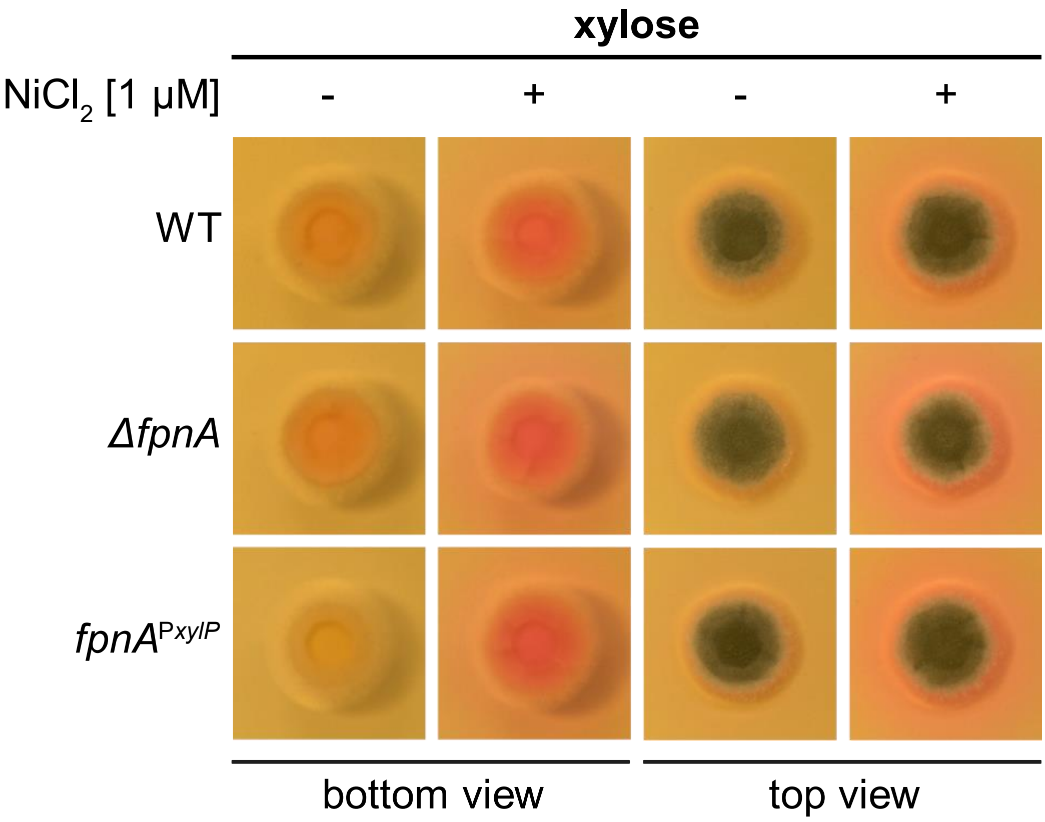

Supplementary Fig. S3 | Nickel supplementation enhances reddish coloration of mycelia (bottom view), which is indicative for increased urease activity and *fpnA* overexpression decreases reddish coloration of mycelia without (-) but not with (+) nickel supplementation. Fungal strains were spot-inoculated on solid minimal medium containing 20 mM urea and 5 mM nitrate as nitrogen sources, 1% xylose for induction of the P*xylP* promoter, 0.012 g/L phenol red as a pH indicator without (-) and with (+) nickel supplementation. Plates were incubated at 37 °C for 72 h. The top view is shown only as a control.

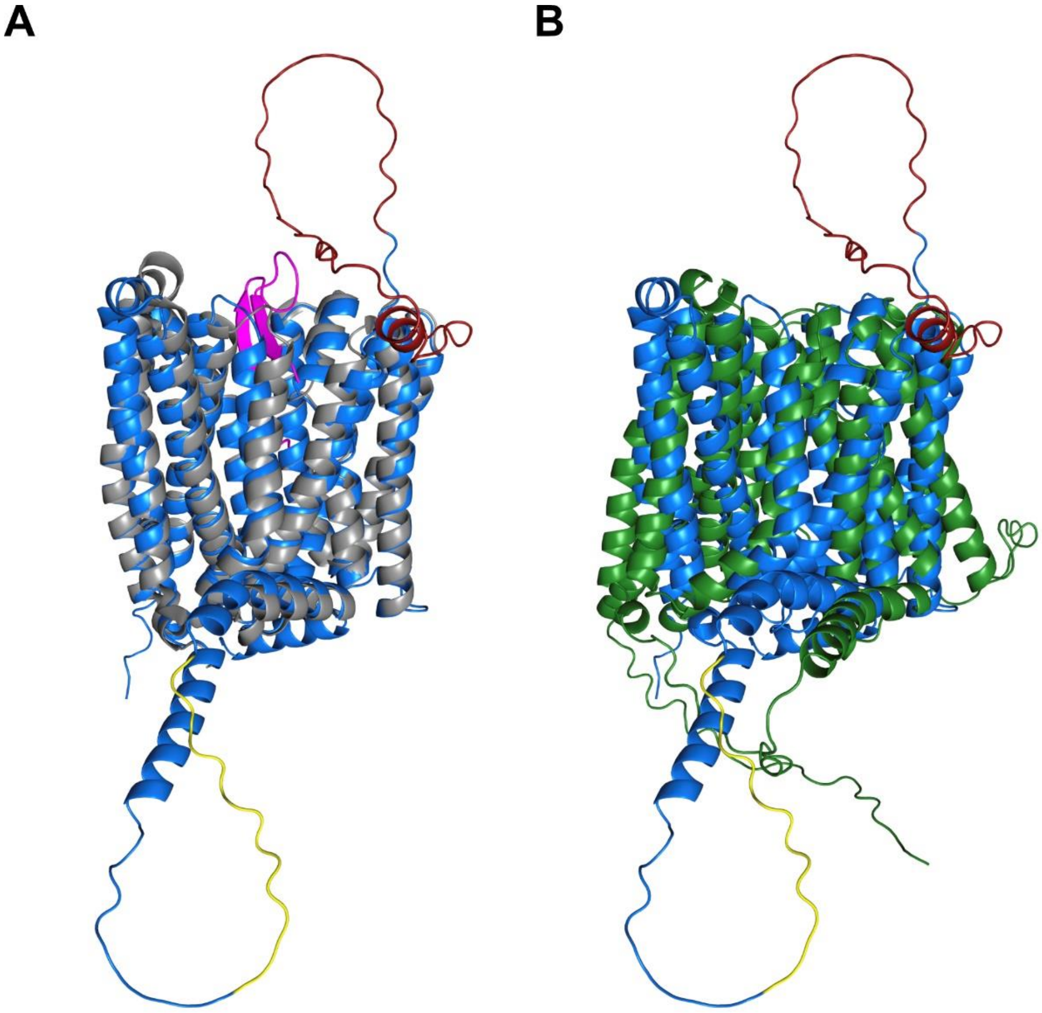

Supplementary Fig. S4 | Structural alignments of Fpn1 and FpnA show high similarities and highlight the protein differences found in two TM loops. (A) Alignment of the human Fpn1 structures from electron microscopy (EM) in gray with the hepcidin ligand (magenta) without assisting Fab45D8 (PDB DOI: 6WBV; https://doi.org/10.2210/pdb6wbv/pdb)[1] and the AlphaFold[2] prediction AF-Q9NP59-F1 in blue: DALI Z-score of 51.3[3]. (B) Structural alignment of the human Fpn1 AlphaFold prediction AF-Q9NP59-F1 (blue) and *A. fumigatus* FpnA calculated with ColabFold[4] (forest-green): DALI Z-score of 28.4; the human Fpn1 EM structure and the *A. fumigatus* FpnA ColabFold prediction show a DALI Z-score of 26.3 (structural alignment not shown). (A and B) The two loops in human Fpn1 that differ significantly compared to the *A. fumigatus* FpnA (also see Fig. 6) are not modelled in the EM structure (6WBV) but in the AlphaFold prediction; the loops are indicated in yellow (between TM6 and TM7: ^{267}GVKDSNIHELEHEQEP^{282}) and in firebrick red (between TM9 and TM10: ^{397}PLDLSVSPFEDIRSRFIQGESITPTKIPEITTEIYMSNGSNSANIVPETSP^{447}).

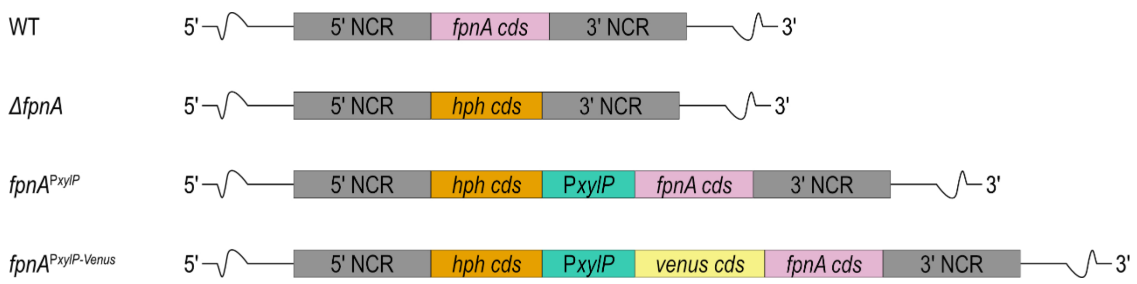

Supplementary Fig. S5 | Schematical illustration of genetic manipulations at the *fpnA* locus in the generated *fpnA* strains.

Supplementary Table S1 | FpnA homologs from selected species included in the phylogenetic analysis (Fig. 5). Given are the corresponding accession numbers from NCBI[5], E-values, query coverage, Bit-scores and taxonomy. The accession number of the query sequence of *Aspergillus fumigatus* FpnA from FungiDB that was used for blastp is highlighted in bold.

accession number	organism	E-Value	query coverage	Bit-score	taxonomy
WP_235046078.1	*Bdellovibrio bacteriovorus*	3.70E-16	86.25	866557	Bacteria; Bdellovibrionota; Bdellovibrionia; Bdellovibrionales; Pseudobdellovibrionaceae
XP_004356781.1	*Acanthamoeba castellanii* str. Neff	1.85E-37	87.03	149443	Eukaryota; Amoebozoa; Discosea; Longamoebia; Centramoebida; Acanthamoebidae
XP_641885.1	*Dictyostelium discoideum* AX4	3.29E-08	86.25	616178	Eukaryota; Amoebozoa; Evosea; Eumycetozoa; Dictyostelia; Dictyosteliales; Dictyosteliaceae
KAJ9461339.1	*Diplonema papillatum*	8.00E-34	88.61	140584	Eukaryota; Discoba; Euglenozoa; Diplonemea; Diplonemidae
KAK6006251.1	*Aureobasidium pullulans*	9.11E-132	88.21	399053	Eukaryota; Fungi; Dikarya; Ascomycota; Pezizomycotina; Dothideomycetes; Dothideomycetidae; Dothideales; Saccotheciaceae
XP_023623651.1	*Ramularia collo-cygni*	1.46E-124	96.66	379407	Eukaryota; Fungi; Dikarya; Ascomycota; Pezizomycotina; Dothideomycetes; Dothideomycetidae; Mycosphaerellales; Mycosphaerellaceae
XP_003857057.1	*Zymoseptoria tritici* IPO323	2.42E-121	88.02	368622	Eukaryota; Fungi; Dikarya; Ascomycota; Pezizomycotina; Dothideomycetes; Dothideomycetidae; Mycosphaerellales; Mycosphaerellaceae
KAI7483109.1	*Hortaea werneckii*	5.75E-128	97.45	390578	Eukaryota; Fungi; Dikarya; Ascomycota; Pezizomycotina; Dothideomycetes; Dothideomycetidae; Mycosphaerellales; Teratosphaeriaceae
KAH8623503.1	*Alternaria alternata*	2.15E-157	93.32	464537	Eukaryota; Fungi; Dikarya; Ascomycota; Pezizomycotina; Dothideomycetes; Pleosporomycetidae; Pleosporales; Pleosporineae; Pleosporaceae
XP_014077774.1	*Bipolaris maydis* ATCC 48331	9.91E-162	94.70	473781	Eukaryota; Fungi; Dikarya; Ascomycota; Pezizomycotina; Dothideomycetes; Pleosporomycetidae; Pleosporales; Pleosporineae; Pleosporaceae
KAF7506784.1	*Endocarpon pusillum*	7.29E-122	95.68	372089	Eukaryota; Fungi; Dikarya; Ascomycota; Pezizomycotina; Eurotiomycetes; Chaetothyriomycetidae; Verrucariales; Verrucariaceae
**Afu5g12920-T**	***Aspergillus fumigatus* Af293**	**-**	**-**	**-**	**Eukaryota; Fungi; Dikarya; Ascomycota; Pezizomycotina; Eurotiomycetes; Eurotiomycetidae; Eurotiales; Aspergillaceae**
GCB19076.1	*Aspergillus awamori*	0	95.68	582408	Eukaryota; Fungi; Dikarya; Ascomycota; Pezizomycotina; Eurotiomycetes; Eurotiomycetidae; Eurotiales; Aspergillaceae
QRD84828.1	*Aspergillus flavus*	0	97.64	678322	Eukaryota; Fungi; Dikarya; Ascomycota; Pezizomycotina; Eurotiomycetes; Eurotiomycetidae; Eurotiales; Aspergillaceae
TPR07554.1	*Aspergillus niger*	0	95.68	627861	Eukaryota; Fungi; Dikarya; Ascomycota; Pezizomycotina; Eurotiomycetes; Eurotiomycetidae; Eurotiales; Aspergillaceae
XP_023092525.1	*Aspergillus oryzae* RIB40	0	92.34	525783	Eukaryota; Fungi; Dikarya; Ascomycota; Pezizomycotina;

					Eurotiomycetes; Eurotiomycetidae; Eurotiales; Aspergillaceae
XP_001211162.1	*Aspergillus terreus* NIH2624	0	96.46	619002	Eukaryota; Fungi; Dikarya; Ascomycota; Pezizomycotina; Eurotiomycetes; Eurotiomycetidae; Eurotiales; Aspergillaceae
XP_001274573.1	*Aspergillus clavatus* NRRL 1	0	98.23	711449	Eukaryota; Fungi; Dikarya; Ascomycota; Pezizomycotina; Eurotiomycetes; Eurotiomycetidae; Eurotiales; Aspergillaceae
XP_664449.1	*Aspergillus nidulans* FGSC A4	0	94.50	590882	Eukaryota; Fungi; Dikarya; Ascomycota; Pezizomycotina; Eurotiomycetes; Eurotiomycetidae; Eurotiales; Aspergillaceae
XP_040661737.1	*Aspergillus versicolor* CBS 583.65	0	89.98	580482	Eukaryota; Fungi; Dikarya; Ascomycota; Pezizomycotina; Eurotiomycetes; Eurotiomycetidae; Eurotiales; Aspergillaceae
CRL27610.1	*Penicillium camemberti*	0	95.09	585489	Eukaryota; Fungi; Dikarya; Ascomycota; Pezizomycotina; Eurotiomycetes; Eurotiomycetidae; Eurotiales; Aspergillaceae
XP_038932385.1	*Penicillium roqueforti*	0	98.23	634024	Eukaryota; Fungi; Dikarya; Ascomycota; Pezizomycotina; Eurotiomycetes; Eurotiomycetidae; Eurotiales; Aspergillaceae
CAP95736.1	*Penicillium rubens* Wisconsin 54-1255	0	93.52	572392	Eukaryota; Fungi; Dikarya; Ascomycota; Pezizomycotina; Eurotiomycetes; Eurotiomycetidae; Eurotiales; Aspergillaceae
XP_054119088.1	*Talaromyces marneffei*	3.42E-123	95.68	374785	Eukaryota; Fungi; Dikarya; Ascomycota; Pezizomycotina; Eurotiomycetes; Eurotiomycetidae; Eurotiales; Trichocomaceae
XP_001248085.2	*Coccidioides immitis* RS	1.15E-123	98.43	375941	Eukaryota; Fungi; Dikarya; Ascomycota; Pezizomycotina; Eurotiomycetes; Eurotiomycetidae; Onygenales; Onygenaceae
KAG7005231.1	*Physcia stellaris*	6.51E-124	96.46	375941	Eukaryota; Fungi; Dikarya; Ascomycota; Pezizomycotina; Lecanoromycetes; OSLEUM clade; Lecanoromycetidae; Caliciales; Physciaceae
SLM34178.1	*Lasallia pustulata*	4.85E-48	84.09	179489	Eukaryota; Fungi; Dikarya; Ascomycota; Pezizomycotina; Lecanoromycetes; OSLEUM clade; Umbilicariomycetidae; Umbilicariales; Umbilicariaceae
XP_024327812.1	*Pseudogymnoascus destructans*	5.64E-129	88.02	389037	Eukaryota; Fungi; Dikarya; Ascomycota; Pezizomycotina; Leotiomycetes; Leotiomycetes incertae sedis; Pseudeurotiaceae
RFU33100.1	*Scytalidium lignicola*	3.07E-46	91.55	174096	Eukaryota; Fungi; Dikarya; Ascomycota; Pezizomycotina; Leotiomycetes; Leotiomycetes incertae sedis
EPS41247.1	*Dactylellina haptotyla* CBS 200.50	3.10E-61	88.02	214927	Eukaryota; Fungi; Dikarya; Ascomycota; Pezizomycotina; Orbiliomycetes; Orbiliales; Orbiliaceae
XP_002837348.1	*Tuber melanosporum*	8.18E-128	91.75	385185	Eukaryota; Fungi; Dikarya; Ascomycota; Pezizomycotina; Pezizomycetes; Pezizales; Tuberaceae
KAG7120527.1	*Verticillium longisporum*	8.31E-115	97.64	35514	Eukaryota; Fungi; Dikarya; Ascomycota; Pezizomycotina; Sordariomycetes; Hypocreomycetidae; Glomerellales; Plectosphaerellaceae
KFH46646.1	*Hapsidospora chrysogenum* ATCC 11550	2.56E-112	89.78	345125	Eukaryota; Fungi; Dikarya; Ascomycota; Pezizomycotina; Sordariomycetes; Hypocreomycetidae; Hypocreales; Bionectriaceae

KID62080.1	*Metarhizium anisopliae* ARSEF 549	7.73E-134	88.21	402134	Eukaryota; Fungi; Dikarya; Ascomycota; Pezizomycotina; Sordariomycetes; Hypocreomycetidae; Hypocreales; Clavicipitaceae
XP_018703135.1	*Cordyceps fumosorosea* ARSEF 2679	5.51E-112	94.89	34551	Eukaryota; Fungi; Dikarya; Ascomycota; Pezizomycotina; Sordariomycetes; Hypocreomycetidae; Hypocreales; Cordycipitaceae
XP_006967377.1	*Trichoderma reesei* QM6a	5.20E-123	87.43	371703	Eukaryota; Fungi; Dikarya; Ascomycota; Pezizomycotina; Sordariomycetes; Hypocreomycetidae; Hypocreales; Hypocreaceae
XP_013946021.1	*Trichoderma atroviride* IMI 206040	1.24E-119	94.70	36477	Eukaryota; Fungi; Dikarya; Ascomycota; Pezizomycotina; Sordariomycetes; Hypocreomycetidae; Hypocreales; Hypocreaceae
XP_011323590.1	*Fusarium graminearum* PH-1	9.21E-117	89.59	357836	Eukaryota; Fungi; Dikarya; Ascomycota; Pezizomycotina; Sordariomycetes; Hypocreomycetidae; Hypocreales; Nectriaceae
XP_018245464.1	*Fusarium oxysporum* f. sp. lycopersici 4287	7.08E-135	89.59	405601	Eukaryota; Fungi; Dikarya; Ascomycota; Pezizomycotina; Sordariomycetes; Hypocreomycetidae; Hypocreales; Nectriaceae
KAJ4225426.1	*Fusarium solani*	1.17E-135	99.02	408683	Eukaryota; Fungi; Dikarya; Ascomycota; Pezizomycotina; Sordariomycetes; Hypocreomycetidae; Hypocreales; Nectriaceae
KAI6351911.1	*Pyricularia grisea*	1.81E-136	92.53	409068	Eukaryota; Fungi; Dikarya; Ascomycota; Pezizomycotina; Sordariomycetes; Sordariomycetidae; Magnaporthales; Pyriculariaceae
XP_961991.3	*Neurospora crassa* OR74A	8.21E-117	92.73	360533	Eukaryota; Fungi; Dikarya; Ascomycota; Pezizomycotina; Sordariomycetes; Sordariomycetidae; Sordariales; Sordariaceae
XP_003348627.1	*Sordaria macrospora* k-hell	1.20E-114	91.16	35514	Eukaryota; Fungi; Dikarya; Ascomycota; Pezizomycotina; Sordariomycetes; Sordariomycetidae; Sordariales; Sordariaceae
KXX75491.1	*Madurella mycetomatis*	7.79E-124	89.98	375941	Eukaryota; Fungi; Dikarya; Ascomycota; Pezizomycotina; Sordariomycetes; Sordariomycetidae; Sordariales; Sordariales incertae sedis
XP_018189894.1	*Xylona heveae* TC161	2.93E-120	95.09	367851	Eukaryota; Fungi; Dikarya; Ascomycota; Pezizomycotina; Xylonomycetes; Xylonales; Xylonaceae
XP_020044921.1	*Ascoidea rubescens* DSM 1968	4.41E-64	89.00	219935	Eukaryota; Fungi; Dikarya; Ascomycota; Saccharomycotina; Saccharomycetes; Saccharomycetales; Ascoideaceae
CDO52397.1	*Geotrichum candidum*	9.88E-111	86.64	340502	Eukaryota; Fungi; Dikarya; Ascomycota; Saccharomycotina; Saccharomycetes; Saccharomycetales; Dipodascaceae
XP_031852041.1	*Saprochaete ingens*	7.05E-104	86.05	324709	Eukaryota; Fungi; Dikarya; Ascomycota; Saccharomycotina; Saccharomycetes; Saccharomycetales; Dipodascaceae
XP_056042578.1	*Lipomyces tetrasporus*	1.72E-127	90.18	385185	Eukaryota; Fungi; Dikarya; Ascomycota; Saccharomycotina; Saccharomycetes; Saccharomycetales; Lipomycetaceae
XP_011271970.1	*Wickerhamomyces ciferrii*	4.87E-53	92.73	19066	Eukaryota; Fungi; Dikarya; Ascomycota; Saccharomycotina; Saccharomycetes;

					Saccharomycetales; Phaffomycetaceae
ODV93542.1	*Pachysolen tannophilus* NRRL Y-2460	8.71E-67	83.50	224942	Eukaryota; Fungi; Dikarya; Ascomycota; Saccharomycotina; Saccharomycetes; Saccharomycetales; Saccharomycetales incertae sedis
ODV88745.1	*Tortispora caseinolytica* NRRL Y-17796	1.51E-70	85.46	234958	Eukaryota; Fungi; Dikarya; Ascomycota; Saccharomycotina; Saccharomycetes; Saccharomycetales; Trigonopsidaceae
KAG2011439.1	*Coprinopsis cinerea* AmutBmut pab1-1	4.95E-47	86.05	176792	Eukaryota; Fungi; Dikarya; Basidiomycota; Agaricomycotina; Agaricomycetes; Agaricomycetidae; Agaricales; Agaricineae; Psathyrellaceae
KAH7343139.1	*Rhizoctonia solani*	1.13E-56	87.03	204912	Eukaryota; Fungi; Dikarya; Basidiomycota; Agaricomycotina; Agaricomycetes; Cantharellales; Ceratobasidiaceae
XP_008032718.1	*Trametes versicolor* FP-101664 SS1	1.47E-68	91.94	233032	Eukaryota; Fungi; Dikarya; Basidiomycota; Agaricomycotina; Agaricomycetes; Polyporales; Polyporaceae
KAF9820241.1	*Postia placenta*	1.20E-75	89.98	251906	Eukaryota; Fungi; Dikarya; Basidiomycota; Agaricomycotina; Agaricomycetes; Polyporales; Postiaceae
KZO98874.1	*Calocera viscosa* TUFC12733	8.93E-59	86.44	205682	Eukaryota; Fungi; Dikarya; Basidiomycota; Agaricomycotina; Dacrymycetes; Dacrymycetales; Dacrymycetaceae
XP_040631131.1	*Dacryopinax primogenitus*	9.85E-56	85.46	199134	Eukaryota; Fungi; Dikarya; Basidiomycota; Agaricomycotina; Dacrymycetes; Dacrymycetales; Dacrymycetaceae
XP_012053293.1	*Cryptococcus neoformans* var. grubii H99	4.76E-63	86.84	218394	Eukaryota; Fungi; Dikarya; Basidiomycota; Agaricomycotina; Tremellomycetes; Tremellales; Cryptococcaceae
XP_014183637.1	*Trichosporon asahii* var. asahii CBS 2479	9.74E-55	89.19	197208	Eukaryota; Fungi; Dikarya; Basidiomycota; Agaricomycotina; Tremellomycetes; Trichosporonales; Trichosporonaceae
KAG0658348.1	*Rhodotorula mucilaginosa*	1.86E-57	86.64	204912	Eukaryota; Fungi; Dikarya; Basidiomycota; Pucciniomycotina; Microbotryomycetes; Sporidiobolales; Sporidiobolaceae
KAE8214764.1	*Tilletia walkeri*	4.76E-51	95.48	187578	Eukaryota; Fungi; Dikarya; Basidiomycota; Ustilaginomycotina; Exobasidiomycetes; Tilletiales; Tilletiaceae
XP_011387882.1	*Ustilago maydis* 521	2.64E-52	87.03	191045	Eukaryota; Fungi; Dikarya; Basidiomycota; Ustilaginomycotina; Ustilaginomycetes; Ustilaginales; Ustilaginaceae
KNE62758.1	*Allomyces macrogynus* ATCC 38327	4.80E-40	92.14	156762	Eukaryota; Fungi; Fungi incertae sedis; Blastocladiomycota; Blastocladiomycetes; Blastocladiales; Blastocladiaceae
TPX56095.1	*Chytriomyces confervae*	1.31E-47	83.30	177178	Eukaryota; Fungi; Fungi incertae sedis; Chytridiomycota; Chytridiomycota incertae sedis; Chytridiomycetes; Chytridiales; Chytriomycetaceae
XP_016610078.1	*Spizellomyces punctatus* DAOM BR117	1.46E-75	95.28	251521	Eukaryota; Fungi; Fungi incertae sedis; Chytridiomycota; Chytridiomycota incertae sedis; Chytridiomycetes; Spizellomycetales; Spizellomycetaceae
EPB87519.1	*Mucor circinelloides* 1006PhL	1.97E-81	88.02	264618	Eukaryota; Fungi; Fungi incertae sedis; Mucoromycota; Mucoromycotina; Mucoromycetes; Mucorales; Mucorineae; Mucoraceae

XP_051459499.1	*Mucor mucedo*	5.23E-74	87.43	244588	Eukaryota; Fungi; Fungi incertae sedis; Mucoromycota; Mucoromycotina; Mucoromycetes; Mucorales; Mucorineae; Mucoraceae
KAG1109301.1	*Rhizopus arrhizus*	1.39E-73	91.16	248054	Eukaryota; Fungi; Fungi incertae sedis; Mucoromycota; Mucoromycotina; Mucoromycetes; Mucorales; Mucorineae; Rhizopodaceae
NP_571704.1	*Danio rerio*	7.51E-41	88.61	16177	Eukaryota; Metazoa; Chordata; Craniata; Vertebrata; Euteleostomi; Actinopterygii; Neopterygii; Teleostei; Ostariophysi; Cypriniformes; Danionidae; Danioninae
AAF44329.1	*Mus musculus*	6.40E-44	87.43	1714	Eukaryota; Metazoa; Chordata; Craniata; Vertebrata; Euteleostomi; Mammalia; Eutheria; Euarchontoglires; Glires; Rodentia; Myomorpha; Muroidea; Muridae; Murinae
NP_055400.1	*Homo sapiens*	2.67E-45	87.03	176022	Eukaryota; Metazoa; Chordata; Craniata; Vertebrata; Euteleostomi; Mammalia; Eutheria; Euarchontoglires; Primates; Haplorrhini; Catarrhini; Hominidae
NP_001032985.1	*Caenorhabditis elegans*	2.97E-18	81.53	939745	Eukaryota; Metazoa; Ecdysozoa; Nematoda; Chromadorea; Rhabditida; Rhabditina; Rhabditomorpha; Rhabditoidea; Rhabditidae; Peloderinae
KAF4039810.1	*Phytophthora infestans*	1.41E-43	94.11	168703	Eukaryota; Sar; Stramenopiles; Oomycota; Peronosporales; Peronosporaceae
XP_012206539.1	*Saprolegnia parasitica* CBS 223.65	1.63E-37	93.71	149828	Eukaryota; Sar; Stramenopiles; Oomycota; Saprolegniales; Saprolegniaceae
NP_001190217.1	*Arabidopsis thaliana*	2.62E-45	89.00	173326	Eukaryota; Viridiplantae; Streptophyta; Embryophyta; Tracheophyta; Spermatophyta; Magnoliopsida; eudicotyledons; Gunneridae; Pentapetalae; rosids; malvids; Brassicales; Brassicaceae; Camelineae

Supplementary Table S2 | Primers used in this study. Small letters represent primer overhangs designed for the construction of the plasmids. PC, plasmid construction; TCA, transformation cassette amplification; NB, Northern blot probe.

primer	sequence [5' → 3']	used for
OH001_pUC19_fwd	GGCATGCAAGCTTGGCGT	PC
OH002_pUC19_rev	GTACCGAGCTCGAATTCACTG	PC
OH003_5'fpnA_fwd	agtgaattcgagctcggtacGATTGCGAGGCTCTCTTTG	PC
OH004_5'fpnA_rev	tagttctgttaccgagccggCACTGGCCCTGATTGCAGT	PC
OH005_hph_fwd	ccggctcggtaacagaactaACGGCGTAACCAAAAGTCAC	PC
OH006_hph_rev	gggagcatatcgttcagagcTCTTGACGACCGTTGATCTG	PC
OH007_3'fpnA_fwd	gctctgaacgatatgctcccCATGATCTATCCAATTTTTATATTATG	PC
OH008_3'fpnA_rev	ttacgccaagcttgcatgccAAAACATGAGTCGCAGCTC	PC
OH009_TCA_fwd	CTTTGAGTCCCTCGTCTGGC	TCA
OH010_TCA_rev	CCGTTGGTCAAATCGAGGTG	TCA
OH011_5'fpnA_rev	tgtacctaggCACTGGCCCTGATTGCAG	PC
OH012_hph_fwd	agggccagtgCCTAGGTACAGAAGTCCAATTG	PC
OH013_hph_rev	tcgcatcagtGTAGGTCTCTTGACGACC	PC
OH014_PxylP_fwd	agagacctacACTGATGCGAGCAACAGTATG	PC
OH015_PxylP_rev	gcgatgccatGGTTGGTTCTTCGAGTCG	PC
OH016_fpnA_cds	agaaccaaccATGGCATCGCAGGTAGATATG	PC
OH017_fpnA_cds_rev	ttacgccaagcttgcatgccCTTTGTAGACAAGCCATCG	PC
OH018_TCA_fwd	TCTGTTCACCTCTTTGAGTCCC	TCA
OH019_TCA_rev	AGCGTCAATCTAACGTGAATAACA	TCA
OH020_PxylP_rev	tgctgaccatGGTTGGTTCTTCGAGTCG	PC
OH021_venus_fwd	agaaccaaccATGGTCAGCAAGGGCGAG	PC
OH022_venus_rev	ggacccggacccggacccCTTGTACAGCTCGTCCATGC	PC
OH023_fpnA_cds_fwd	gggtccgggtccgggtccATGGCATCGCAGGTAGATATG	PC
OH024_fpnA_fwd	ATGGCATCGCAGGTAGATATG	NB
OH025_fpnA_rev	CTTTGTAGACAAGCCATCGAC	NB
OH026_cccA_fwd	GATTCCGACACCCTAGAC	NB
OH027_cccA_rev	GCGATGATGTTGTCCCTG	NB
OH028_gpdA_fwd	ACGACCAGGGTCTGATTGTC	NB
OH029_gpdA_rev	CTTGATGGCCTGCTTGATCT	NB

Supplementary Table S3 | Strains used in this study.

strain	description	Reference
AfS77 (WT)	ATCC4664; *ΔakuA::loxP*	[6]
ΔfpnA	AfS77; *ΔfpnA::hph*	this study
fpnA^{PxylP}	AfS77; 5'*fpnA::hph*, P*xylP:fpnA*	this study
fpnA$^{PxylP\text{-}Venus}$	AfS77; 5'*fpnA::hph*, P*xylP:Venus:fpnA*	this study
ΔsreA	AfS77; *ΔsreA::ptrA*	[7]
ΔfpnAΔsreA	AfS77; *ΔfpnA::hph, ΔsreA::ptrA*	this study
fpnA^{PxylP}*ΔsreA*	AfS77; 5'*fpnA::hph*, P*xylP:fpnA, ΔsreA::ptrA*	this study

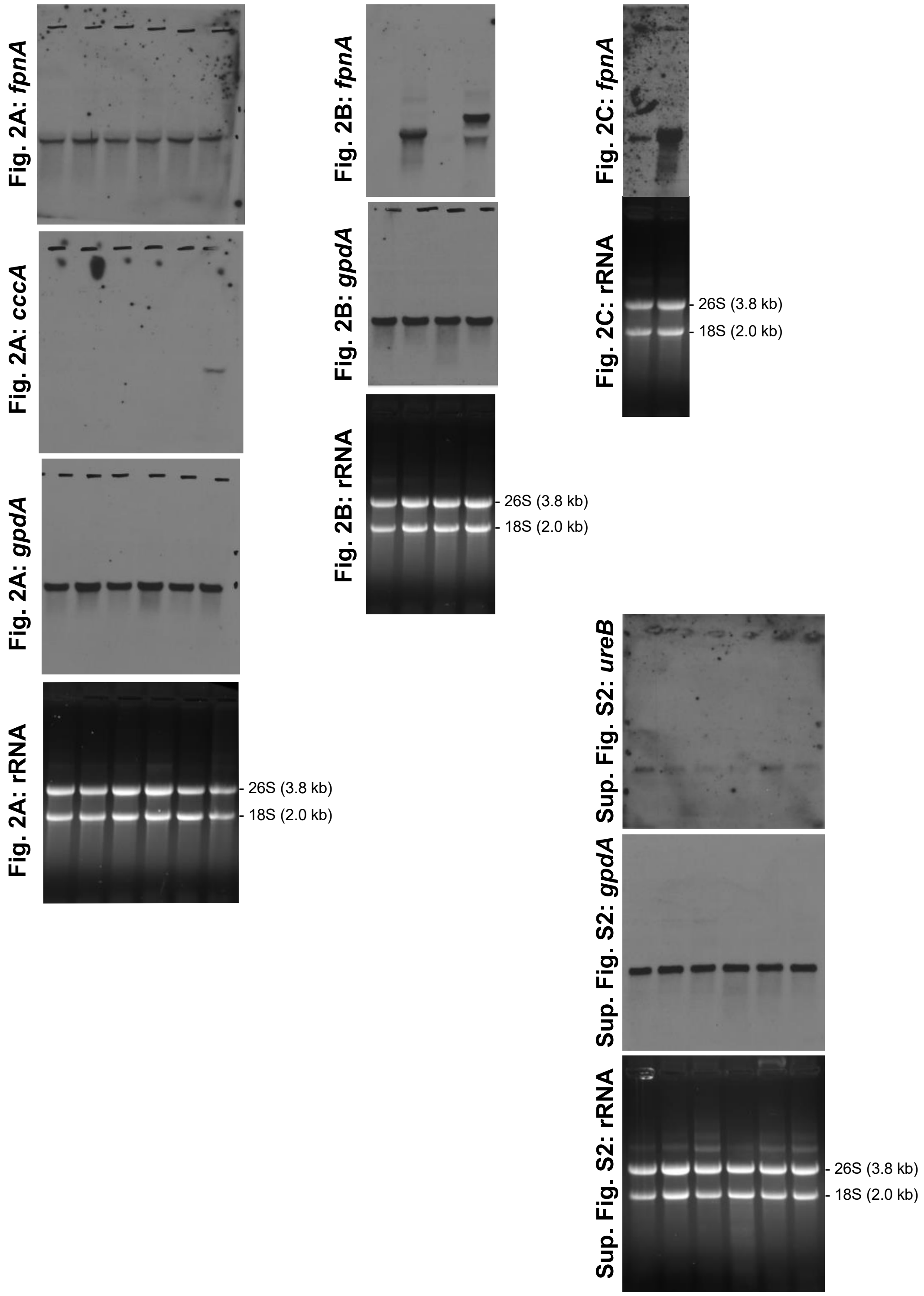

Supplementary Fig. S6 | Uncropped blot images from Fig. 2 and Supplementary Fig. S2.

Supplementary References

1. Billesbølle, C. B. *et al.* Structure of hepcidin-bound ferroportin reveals iron homeostatic mechanisms. *Nature* **586**, 807–811 (2020).
2. Varadi, M. *et al.* AlphaFold Protein Structure Database: massively expanding the structural coverage of protein-sequence space with high-accuracy models. *Nucleic Acids Research* **50**, D439–D444 (2022).
3. Holm, L., Laiho, A., Törönen, P. & Salgado, M. DALI shines a light on remote homologs: One hundred discoveries. *Protein Science* **32**, e4519 (2023).
4. Mirdita, M. *et al.* ColabFold: making protein folding accessible to all. *Nat Methods* **19**, 679–682 (2022).
5. Sayers, E. W. *et al.* Database resources of the National Center for Biotechnology Information. *Nucleic Acids Research* **47**, D23–D28 (2019).
6. Hartmann, T. *et al.* Validation of a self-excising marker in the human pathogen Aspergillus fumigatus by employing the beta-rec/six site-specific recombination system. *Appl Environ Microbiol* **76**, 6313–6317 (2010).
7. Misslinger, M. *et al.* The monothiol glutaredoxin GrxD is essential for sensing iron starvation in Aspergillus fumigatus. *PLoS Genet* **15**, e1008379 (2019).
